# Person-Centered vs Persons-Centered: Exploring Quality-of-Life Promotion Among Administrators in Nursing Homes

**DOI:** 10.1093/geroni/igaf122.696

**Published:** 2025-12-31

**Authors:** Olivia Black, Jing Wang

**Affiliations:** Boston College, Hampton, New Hampshire, United States; University of New Hampshire, Durham, New Hampshire, United States

## Abstract

As dementia is a progressive and incurable condition, improving QoL stands as a paramount objective in care. While there are strategies to improve QoL for persons with dementia (PWD), person-centered care is particularly prominent. However, defining QoL for PWD is complex; it is widely acknowledged to be subjective and multifaceted. There is a notable gap in research regarding how PWD themselves, as well as those who care for and support them, perceive QoL. Our study investigates general perceptions of QoL and specifically how it is understood and promoted for PWD in nursing homes, as described by nursing home administrators (i.e. executive directors, therapeutic directors). We utilized an exploratory and descriptive qualitative study design, gathering data through semi-structured interviews with thirteen administrators from a variety of nursing homes, which ranged from independent to chain-affiliated facilities that accommodate PWD. Results demonstrate a divergence in perspectives, with some interviewees stating that QoL is tied to social relationships, whereas others stating that QoL is more about preserving individual agency. Results also revealed a divergence in prioritization among administrators, with some emphasizing person-centered QoL measures while others focused on broader QoL promotion for the resident community. Additionally, interviewees highlighted variations in the conceptualization of QoL across micro-communities within the facility, particularly in facilities with separate memory care units. Moreover, some administrators expressed challenges in maintaining consistent practices of QoL promotion due to the changing needs of residents. These findings have significant implications for the conceptualization and development of person-centered QoL measures.

